# Supplementary material for: Infections in Glucose-6-Phosphate Dehydrogenase G6PD-Deficient Patients; Predictors for Infection-Related Mortalities and Treatment Outcomes
Source: Antibiotics (Basel). 2023 Mar 1;12(3):494. doi: 10.3390/antibiotics12030494 (PMC10044656; doi:10.3390/antibiotics12030494)
Supplement: Supplementary file 1 [file antibiotics-12-00494-s001.zip › antibiotics-2143174-supplementary.pdf]

Supplementary Materials:

Table S1. Patient's Demographics.

		Overall N (%)		Recovery N (%)		Death N (%)	P value Chi
Gender	Female	123 (32)		82 (29.9)		32 (30.5)	1.000
	Male	256 (68)		192 (70.1)		73 (69.5)	
Age	median (IQR)	60 (41-77)		53 (35-72)		77 (68-80)	<0.001
	⩽ 60 Years	190 (50)		172 (62.8)		18 (17.1)	<0.001
	> 60 years	189 (50)		102 (37.2)		87 (82.9)	
Length of stay	median (IQR)	12 (5-31)		9 (5-24)		26 (13-43)	<0.001
	⩽ 14 days	195 (51)		168 (61.3)		27 (25.7)	<0.001
	> 14 days	184 (49)		106 (38.7)		78 (74.3)	
Admission with infectious disease	No	105 (29)		91 (33.2)		15 (14.3)	
	Yes	273 (72)		183 (66.8)		90 (85.7)	<0.001
Admission to critical care area	No	262 (69)		217 (79.2)		45 (42.9)	<0.001
	Yes	117 (31)		57 (20.8)		60 (57.1)	
Underlying Comorbidities							
Diabetes	No	125 (33)		104 (38.0)		21 (20.0)	0.001
	Yes	254 (67)		170 (62.0)		84 (80.0)	
Chronic renal failure	No	151 (40)		133 (48.5)		18 (17.1)	<0.001
	Yes	228 (60)		141 (51.5)		87 (82.9)	
Active malignancy	No	365 (96)		267 (97.4)		98 (93.3)	0.111
	Yes	14 (4)		7 (2.6)		7 (6.7)	
Immunosuppressed	No	355 (94)		259 (94.5)		96 (91.4)	0.383
	Yes	24 (6)		15 (5.5)		9 (8.6)	
Chronic Cardiac Diseases	No	97 (26)		89 (32.5)		8 (7.6)	<0.001
	Yes	282 (74)		185 (67.5)		97 (92.4)	
HIV follow-up AIDS	No	278 (100)		274 (100.0)		104 (99.0)	0.618
	Yes	1 (0)		0 (0.0)		1 (1.0)	
Chronic Resp. Disease	No	309 (82)		247 (90.1)		62 (59.0)	<0.001
	Yes	70 (18)		27 (9.9)		43 (41.0)	
Sickle Cell	No	359 (95)		256 (93.4)		104 (99.0)	0.048
	Yes	19 (5)		18 (6.6)		1 (1.0)	
Others	No	200 (53)		152 (55.5)		48 (45.7)	0.112
	Yes	179 (47)		122 (44.5)		57 (54.3)	
Comorbid conditions	No. median (IQR)	3 (2-4)		4 (3-4)		3 (2-4)	<0.001
	No comorbidity	41 (11)		40 (14.6)		1 (1.0)	<0.001

	Any comorbidity	338 (89)		234 (85.4)		104 (99.0)	
Risk Factors for infection							
Blood transfusion during admission	No	167 (44)		146 (53.3)		21 (20.0)	<0.001
	Yes	212 (56)		128 (46.7)		84 (80.0)	
Invasive procedure during admission	No	94 (25)		92 (33.6)		2 (1.9)	<0.001
	Yes	285 (75)		182 (66.4)		103 (98.1)	
Surgery 90-day history	No	340 (90)		239 (87.2)		101 (96.2)	0.017
	Yes	39 (10)		35 (12.8)		4 (3.8)	
Type of infection							
Bacteremia	No	291 (77)		218 (79.6)		73 (69.5)	0.053
	Yes	88 (23)		56 (20.4)		32 (30.5)	
Body Fluids	No	378 (100)		273 (99.6)		105 (100.0)	1.000
	Yes	1 (0)		1 (0.4)		0 (0.0)	
Respiratory infections	No	288 (76)		221 (80.7)		67 (63.8)	0.001
	Yes	91 (24)		53 (19.3)		38 (36.2)	
Skin and soft tissue infections	No	276 (73)		188 (68.6)		88 (83.8)	0.004
	Yes	103 (27)		86 (31.4)		17 (16.2)	
Urinary tract infections	No	283 (75)		196 (71.5)		87 (82.9)	0.033
	Yes	96 (25)		78 (28.5)		18 (17.1)	
Gram-negative infections	No	152 (40)		111 (40.5)		41 (39.0)	0.886
	Yes	227 (60)		163 (59.5)		64 (61.0)	
Gram-positive infections	No	272 (72)		194 (70.8)		78 (74.3)	0.585
	Yes	107 (28)		80 (29.2)		27 (25.7)	
Fungal infections	No	348 (92)		252 (92.0)		96 (91.4)	1.000
	Yes	31 (8)		22 (8.0)		9 (8.6)	
SARS-CoV19 infections	No	365 (96)		265 (96.7)		100 (95.2)	0.705
	Yes	14 (4)		9 (3.3)		5 (4.8)	
CRE infections	No	255 (94)		253 (92.3)		102 (97.1)	0.138
	Yes	24 (6)		21 (7.7)		3 (2.9)	
ESBL infections	No	329 (87)		239 (87.2)		90 (85.7)	0.826
	Yes	50 (13)		35 (12.8)		15 (14.3)	
MDR infections	No	312 (82)		235 (85.8)		77 (73.3)	0.007
	Yes	67 (18)		39 (14.2)		28 (26.7)	
MRSA infections	No	367 (97)		263 (96.0)		104 (99.0)	0.232
	Yes	12 (3)		11 (4.0)		1 (1.0)	
Resistant phenotypes	No	226 (60)		168 (61.3)		58 (55.2)	0.336
	Yes	153 (40)		106 (38.7)		47 (44.8)	

Place of Acquisition	Community	212 (56)		180 (65.7)		32 (30.5)		<0.001
	Hospital	167 (44)		94 (34.3)		73 (69.5)		
90-day recurrence of any infection	No	243 (64)		143 (52.2)		100 (95.2)		<0.001
	Yes	136 (36)		131 (47.8)		5 (4.8)		
Antimicrobial treatment								
Cephalosporins (90-day exposure)	No	273 (82)		198 (72.3)		76 (72.4)		1.000
	Yes	105 (28)		76 (27.7)		29 (27.6)		
Aminoglycosides (90-day exposure)	No	354 (93)		253 (92.3)		101 (96.2)		0.262
	Yes	25 (7)		21 (7.7)		4 (3.8)		
Tetracyclines (90-day exposure)	No	360 (95)		259 (94.5)		101 (96.2)		0.688
	Yes	19 (5)		15 (5.5)		4 (3.8)		
B-lactam/B-lactamase (90-day exposure)	No	321 (85)		242 (88.3)		79 (75.2)		0.003
	Yes	58 (15)		32 (11.7)		26 (24.8)		
Macrolides (90-day exposure)	No	345 (91)		258 (94.2)		87 (82.9)		0.001
	Yes	34 (9)		16 (5.8)		18 (17.1)		
Glycopeptides (90-day exposure)	No	346 (91)		245 (89.4)		101 (96.2)		0.059
	Yes	33 (9)		29 (10.6)		4 (3.8)		
Nitroimidazole (90-day exposure)	No	350 (92)		256 (93.4)		94 (89.5)		0.287
	Yes	29 (8)		18 (6.6)		11 (10.5)		
Colistin (90-day exposure)	No	378 (100		273 (99.6)		105 (100.0)		1.000
	Yes	1 (0)		1 (0.4)		0 (0.0)		
B-lactams (90-day exposure)	No	310 (82)		232 (84.7)		77 (73.3)		0.016
	Yes	70 (18)		42 (15.3)		28 (26.7)		
Glycylcycline (90-day exposure)	No	376 (99)		271 (98.9)		105 (100.0)		0.668
	Yes	3 (1)		3 (1.1)		0 (0.0)		
Quinolones (90-day exposure)	No	315 (83)		225 (82.1)		90 (85.7)		0.494
	Yes	64 (17)		49 (17.9)		15 (14.3)		
Oxazolidinones (90-day exposure)	No	377 (99)		272 (99.3)		105 (100.0)		0.932
	Yes	2 (1)		2 (0.7)		0 (0.0)		
90-day exposure to any antibiotic	No	221 (58)		166 (60.6)		55 (52.4)		0.182
	Yes	158 (42)		108 (39.4)		50 (47.6)		
History of infection								
90-days prior infection	No	292 (77)		211 (77.0)		81 (77.1)		1.000
	Yes	87 (23)		63 (23.0)		24 (22.9)		
Previous Gram-negative infection	No	321 (87)		233 (85.0)		98 (93.3)		0.045
	Yes	48 (13)		41 (15.0)		7 (6.7)		
Previous Gram-positive infection	No	345 (91)		242 (88.3)		103 (98.1)		0.005

	Yes	34 (9)		32 (11.7)		2 (1.9)		
Previous fungal infections	No	375 (99)		270 (98.5)		105 (100.0)		0.495
	Yes	4 (1)		4 (1.5)		0 (0.0)		
Previous SARSCoV19 infection	No	259 (95)		271 (98.9)		88 (83.8)		<0.001
	Yes	20 (5)		3 (1.1)		17 (16.2)		
Polymicrobial Infections	No	155 (41)		137 (50.0)		18 (17.1)		<0.001
	Yes	224 (59)		137 (50.0)		87 (82.9)		
Concurrent Gram-negative infection	No	210 (55)		176 (64.2)		34 (32.4)		<0.001
	Yes	169 (45)		98 (35.8)		71 (67.6)		
Concurrent Gram-positive infection	No	273 (72)		206 (75.2)		67 (63.8)		0.038
	Yes	106 (28)		68 (24.8)		38 (36.2)		
Concurrent Fungal infection	No	320 (84)		249 (90.9)		71 (67.6)		<0.001
	Yes	59 (16)		25 (9.1)		34 (32.4)		
Concurrent SARSCoV19 infection	No	372 (98)		274 (100.0)		98 (93.3)		<0.001
	Yes	7 (2)		0 (0.0)		7 (6.7)		

Table S2. Predictors for infection-related crude all-cause in-hospital mortality.

		Recovery n (%)	Death n (%)	Univariable OR (CI, P)		OR (multivariable)
Sex	Female	82 (71.9)	32 (28.1)	-		
	Male	192 (72.5)	73 (27.5)	0.97 (0.6-1.6, p=0.917)		
Age on admission	Age Mean (SD)	53.2 (21.2)	72.9 (12.7)	1.1 (1.0-1.1, p<0.001)	#	1.13 (1.05-1.24, p=0.003)
	Age ≤ 60 Years	172 (90.5)	18 (9.5)	-		
	Age > 60 years	102 (54.0)	87 (46.0)	8.2 (4.7-14.7, p<0.001)	#	3.00 (0.20-45.77, p=0.426)
Length of stay	LOS Mean (SD)	17.7 (19.2)	46. (48.7)	1.03 (1.02-1.04, p<0.001)	#	0.98 (0.96-1.02, p=0.321)
	LOS ≤ 14 days	168 (86.2)	27 (13.8)	-		
	LOS > 14 days	106 (57.6)	78 (42.4)	4.58 (2.81-7.66, p<0.001)	#	0.05 (0.01-0.35, p=0.004)
Admission with infectious disease	No	91 (85.8)	15 (14.2)	-		
	Yes	183 (67.0)	90 (33.0)	2.98 (1.68-5.63, p<0.001)	#	14.59 (2.0-156.98, p=0.015)
Admission to critical care area	No	217 (82.8)	45 (17.2)	-		
	Yes	57 (48.7)	60 (51.3)	5.08 (3.14-8.29, p<0.001)	#	46.39 (5.1-716.25, p=0.002)
Diabetes	No	104 (83.2)	21 (16.8)	-		
	Yes	170 (66.9)	84 (33.1)	2.45 (1.45-4.27, p=0.001)	#	0.03 (0.00-0.48, p=0.077)
Chronic renal failure	No	133 (88.1)	18 (11.9)	-		
	Yes	141 (61.8)	87 (38.2)	4.56 (2.66-8.19, p<0.001)	#	0.12 (0.00-2.51, p=0.328)
Active malignancy	No	267 (73.2)	98 (26.8)	-		
	Yes	7 (50.0)	7 (50.0)	2.72 (0.91-8.15, p=0.067)	#	0.11 (0.00-71.31, p=0.598)
Immunosuppressed	No	259 (73.0)	96 (27.0)	-		
	Yes	15 (62.5)	9 (37.5)	1.62 (0.66-3.76, p=0.272)		
Chronic Cardiac Diseases	No	89 (91.8)	8 (8.2)	-		
	Yes	185 (65.6)	97 (34.4)	5.83 (2.87-13.5, p<0.001)	#	0.15 (0.00-6.99, p=0.423)
Chronic Cardiac Diseases	No	274 (72.5)	104 (27.5)	-		
	Yes	0 (0.0)	1 (100.0)	*(p=0.978)		
Chronic Resp. Disease	No	247 (79.9)	62 (20.1)	-		
	Yes	27 (38.6)	43 (61.4)	6.34 (3.66-11.2, p<0.001)	#	1.10 (0.00-29.04, p=0.966)
Sickle Cell	No	256 (71.1)	104 (28.9)	-		
	Yes	18 (94.7)	1 (5.3)	0.14 (0.01-0.68, p=0.054)	#	0.01 (0.00-0.87, p=0.081)
Other comorbidities	No	152 (76.0)	48 (24.0)	-		
	Yes	122 (68.2)	57 (31.8)	1.48 (0.94-2.33, p=0.089)	#	0.36 (0.00-7.60, p=0.626)
Comorbid conditions	Mean (SD)	3.5 (1.4)	4.7 (1.1)	2.06 (1.67-2.58, p<0.001)	#	6.11 (0.36-2119.6, p=0.358)
	No comorbidity	40 (97.6)	1 (2.4)	-		
	Any comorbidity	234 (69.2)	104 (30.8)	17.8 (3.8-317.5, p=0.005)	#	111.3 (1.3-15131, p=0.045)
Blood transfusion during admission	No	146 (87.4)	21 (12.6)	-		
	Yes	128 (60.4)	84 (39.6)	4.56 (2.72-7.94, p<0.001)	#	0.95 (0.21-4.00, p=0.942)
Invasive procedure during admission	No	92 (97.9)	2 (2.1)	-		
	Yes	182 (63.9)	103 (36.1)	26.03 (8-160.03, p<0.001)	#	392.6 (37.8-7562, p<0.001)
Surgery 90-day history	No	239 (70.3)	101 (29.7)	-		
	Yes	35 (89.7)	4 (10.3)	0.27 (0.08-0.70, p=0.016)	#	0.11 (0.01-0.73, p=0.031)
Bacteremia	No	218 (74.9)	73 (25.1)	-		
	Yes	56 (63.6)	32 (36.4)	1.71 (1.02-2.83, p=0.040)	#	* p=0.992
Body Fluids	No	273 (72.2)	105 (27.8)	-		
	Yes	1 (100.0)	0 (0.0)	(p=0.981)		
Respiratory infections	No	221 (76.7)	67 (23.3)	-		
	Yes	53 (58.2)	38 (41.8)	2.36 (1.43-3.89, p=0.001)	#	*p=0.992
Skin and soft tissue infections	No	188 (68.1)	88 (31.9)	-		
	Yes	86 (83.5)	17 (16.5)	0.42 (0.23-0.74, p=0.003)	#	*p=0.993
Urinary tract infections	No	196 (69.3)	87 (30.7)			
	Yes	78 (81.2)	18 (18.8)	0.52 (0.29, 0.92, P=0.025)	#	* p=0.993
Gram-negative infections	No	111 (73.0)	41 (27.0)	-		
	Yes	163 (71.8)	64 (28.2)	1.06 (0.67-1.69, p=0.795)		
Gram-positive infections	No	194 (71.3)	78 (28.7)	-		
	Yes	80 (74.8)	27 (25.2)	0.84 (0.50-1.38, p=0.501)		
Fungal infections	No	252 (72.4)	96 (27.6)	-		
	Yes	22 (71.0)	9 (29.0)	1.07 (0.45-2.34, p=0.863)		
CRE infections	No	253 (71.3)	102 (28.7)	-		
	Yes	21 (87.5)	3 (12.5)	0.35 (0.08-1.06, p=0.099)	#	0.02 (0.00-0.39, p=0.013)
ESBL infections	No	239 (72.6)	90 (27.4)	-		
	Yes	35 (70.0)	15 (30.0)	1.14 (0.58-2.15, p=0.697)		
MDR infections	No	235 (75.3)	77 (24.7)	-		
	Yes	39 (58.2)	28 (41.8)	2.19 (1.26-3.79, p=0.005)	#	0.64 (0.13-3.11, p=0.575)
MRSA infections	No	263 (71.7)	104 (28.3)	-		
	Yes	11 (91.7)	1 (8.3)	0.23 (0.01-1.20, p=0.162)	#	1.36 (0.04-19.46, p=0.840)
Place of Acquisition	Community	180 (84.9)	32 (15.1)	-		
	Hospital	94 (56.3)	73 (43.7)	4.37 (2.71-7.17, p<0.001)	#	5.22 (1.13-28.78, p=0.043)
	No	198 (72.3)	76 (27.7)	-		

Cephalosporins (90-day exposure)	Yes	76 (72.4)	29 (27.6)	0.99 (0.60-1.63, p=0.982)		
Aminoglycosides (90-day exposure)	No	253 (71.5)	101 (28.5)	-		
	Yes	21 (84.0)	4 (16.0)	0.48 (0.14-1.29, p=0.185)	#	2.17 (0.02-210.65, p=0.739)
Tetracyclines (90-day exposure)	No	259 (71.9)	101 (28.1)	-		
	Yes	15 (78.9)	4 (21.1)	0.68 (0.19-1.94, p=0.509)		
B-lactam/B-lactamase (90-day exposure)	No	242 (75.4)	79 (24.6)	-		
	Yes	32 (55.2)	26 (44.8)	2.49 (1.39-4.43, p=0.002)	#	141.9 (10.7-2759, p<0.001)
Macrolides (90-day exposure)	No	258 (74.8)	87 (25.2)	-		
	Yes	16 (47.1)	18 (52.9)	3.34 (1.63-6.89, p=0.001)	#	1.68 (0.09-31.01, p=0.724)
Glycopeptides (90-day exposure)	No	245 (70.8)	101 (29.2)	-		
	Yes	29 (87.9)	4 (12.1)	0.33 (0.10-0.88, p=0.045)	#	0.07 (0.00-2.54, p=0.171)
Nitroimidazole (90-day exposure)	No	256 (73.1)	94 (26.9)	-		
	Yes	18 (62.1)	11 (37.9)	1.66 (0.74-3.61, p=0.204)	#	39.83 (1.2-1506.5, p=0.043)
Colistin (90-day exposure)	No	273 (72.2)	105 (27.8)	-		
	Yes	1 (100.0)	0 (0.0)	0.00 (*, p=0.981)		
B-lactams (90-day exposure)	No	232 (75.1)	77 (24.9)	-		
	Yes	42 (60.0)	28 (40.0)	2.01 (1.16-3.45, p=0.012)	#	15.13 (1.57-172.3, p=0.021)
Quinolones (90-day exposure)	No	225 (71.4)	90 (28.6)	-		
	Yes	49 (76.6)	15 (23.4)	0.77 (0.40-1.40, p=0.404)		
Oxazolidinones (90-day exposure)	No	272 (72.1)	105 (27.9)	-		
	Yes	2 (100.0)	0 (0.0)	0.00 (*, p=0.983)		
90-day exposure to any antibiotic	No	166 (75.1)	55 (24.9)	-		
	Yes	108 (68.4)	50 (31.6)	1.40 (0.89-2.20, p=0.148)	#	0.28 (0.02-3.55, p=0.330)
90-days prior infection	No	211 (72.3)	81 (27.7)	-		
	Yes	63 (72.4)	24 (27.6)	0.99 (0.57-1.68, p=0.978)		
Previous Gram-negative infection	No	233 (70.4)	98 (29.6)	-		
	Yes	41 (85.4)	7 (14.6)	0.41 (0.16-0.88, p=0.034)	#	0.72 (0.04-9.96, p=0.812)
Previous Gram-positive infection	No	242 (70.1)	103 (29.9)	-		
	Yes	32 (94.1)	2 (5.9)	0.15 (0.02-0.50, p=0.009)	#	0.01 (0.00-0.68, p=0.052)
Previous fungal infection	No	270 (72.0)	105 (28.0)	-		
	Yes	4 (100.0)	0 (0.0)	*p=0.984		
Previous SARSCoV19 infection	No	271 (75.5)	88 (24.5)	-		
	Yes	3 (15.0)	17 (85.0)	17.45 (5.70-76, p<0.001)	#	1.42 (0.01-123.01, p=0.873)
Polymicrobial Infections	No	137 (88.4)	18 (11.6)	-		
	Yes	137 (61.2)	87 (38.8)	4.83 (2.82-8.69, p<0.001)	#	1.91 (0.19-19.60, p=0.581)
Concurrent Gram-negative infection	No	176 (83.8)	34 (16.2)	-		
	Yes	98 (58.0)	71 (42.0)	3.75 (2.34-6.10, p<0.001)	#	3.03 (0.51-21.84, p=0.241)
Concurrent Gram-positive infection	No	206 (75.5)	67 (24.5)	-		
	Yes	68 (64.2)	38 (35.8)	1.72 (1.06-2.78, p=0.028)	#	0.71 (0.15-3.24, p=0.652)
Concurrent Fungal infection	No	249 (77.8)	71 (22.2)	-		
	Yes	25 (42.4)	34 (57.6)	4.77 (2.68-8.59, p<0.001)	#	1.73 (0.25-12.42, p=0.575)
Concurrent SARSCoV19 infection	No	274 (73.7)	98 (26.3)	-		
	Yes	0 (0.0)	7 (100.0)	*p=0.976		

#: variables with p-values ≤ 0.2 are enrolled in multivariate analysis, *: value is too high/too low to be detected by the software, 95% CI: Confidence intervals.

Table S3. Predictors for infection-related 14-day in-hospital mortality. 10

		No	Yes	Univariable OR (CI, P)		Multivariable OR (CI, P)
Sex	F	111 (97.4)	3 (2.6)	-		
	M	241 (90.9)	24 (9.1)	3.68 (1.25-15.73, p=0.036)	#	5.01 (0.70-65.98, p=0.154)
Age on admission.	Age Mean (SD)	57.5 (21.1)	73.4 (16.0)	1.05 (1.02-1.07, p<0.001)	#	1.06 (0.99-1.14, p=0.116)
	Age ≤ 60 Years	185 (97.4)	5 (2.6)	-		
	Age > 60 years	167 (88.4)	22 (11.6)	4.87 (1.95-14.81, p=0.002)	#	1.06 (0.08-15.47, p=0.967)
Length of stay	Mean (SD)	27.2 (33.7)	5.3 (3.7)	0.85 (0.77-0.92, p<0.001)	#	0.71 (0.58-0.83, p<0.001)
	LOS ≤ 14 days	168 (86.2)	27 (13.8)	-		
	LOS > 14 days	184 (100.0)	0 (0.0)	* (p=0.989)		
Admission with infectious disease	No	100 (94.3)	6 (5.7)	-		
	Yes	252 (92.3)	21 (7.7)	1.39 (0.58-3.88, p=0.492)		
Admission to critical care area	No	246 (93.9)	16 (6.1)	-		
	Yes	106 (90.6)	11 (9.4)	1.60 (0.70-3.52, p=0.253)		
Admission to medical wards	No	220 (94.4)	13 (5.6)	-		
	Yes	132 (90.4)	14 (9.6)	1.79 (0.81-3.98, p=0.144)		
Diabetes	No	115 (92.0)	10 (8.0)	-		
	Yes	237 (93.3)	17 (6.7)	0.82 (0.37-1.92, p=0.642)		
Chronic renal failure	No	144 (95.4)	7 (4.6)	-		
	Yes	208 (91.2)	20 (8.8)	1.98 (0.85-5.15, p=0.132)	#	0.60 (0.11-3.20, p=0.550)
Active malignancy	No	339 (92.9)	26 (7.1)	-		
	Yes	13 (92.9)	1 (7.1)	1.00 (0.05-5.35, p=0.998)		
Immunosuppressed	No	330 (93.0)	25 (7.0)	-		
	Yes	22 (91.7)	2 (8.3)	1.20 (0.19-4.40, p=0.812)		
Chronic Cardiac Diseases	No	92 (94.8)	5 (5.2)	-		
	Yes	260 (92.2)	22 (7.8)	1.56 (0.62-4.75, p=0.385)		
HIV follow up	No	352 (93.1)	26 (6.9)	-		
	Yes	0 (0.0)	1 (100.0)	* (p=0.984)		
Chronic Resp. Disease	No	287 (92.9)	22 (7.1)	-		
	Yes	65 (92.9)	5 (7.1)	1.00 (0.33-2.56, p=0.995)		
Sickle Cell	No	333 (92.5)	27 (7.5)	-		
	Yes	19 (100.0)	0 (0.0)	* (p=0.987)		
Other comorbidities	No	186 (93.0)	14 (7.0)	-		
	Yes	166 (92.7)	13 (7.3)	1.04 (0.47-2.29, p=0.921)		
Comorbid conditions	Mean (SD)	3.8 (1.5)	4.0 (0.9)	1.10 (0.84-1.46, p=0.511)		
	No comorbid	40 (97.6)	1 (2.4)	-		
	Any comorbid	312 (92.3)	26 (7.7)	3.33 (0.68-60.30, p=0.244)		
Blood transfusion during admission	No	151 (90.4)	16 (9.6)	-		
	Yes	201 (94.8)	11 (5.2)	0.52 (0.23-1.14, p=0.104)	#	1.73 (0.43-7.20, p=0.441)
Invasive procedure during admission	No	92 (97.9)	2 (2.1)	-		
	Yes	260 (91.2)	25 (8.8)	4.42 (1.28-27.83, p=0.046)	#	100.8 (13.88-1455.8, p<0.001)
Surgery 90-day history	No	313 (92.1)	27 (7.9)	-		
	Yes	39 (100.0)	0 (0.0)	* (p=0.988)		
Bacteremia	No	277 (95.2)	14 (4.8)	-		
	Yes	75 (85.2)	13 (14.8)	3.43 (1.53-7.65, p=0.002)	#	2.06 (0.40-11.46, p=0.389)
Body Fluids	No	351 (92.9)	27 (7.1)	-		
	Yes	1 (100.0)	0 (0.0)	* (p=0.989)		
Respiratory infections	No	266 (92.4)	22 (7.6)	-		
	Yes	86 (94.5)	5 (5.5)	0.70 (0.23-1.78, p=0.490)		
Skin and soft tissue infections	No	253 (91.7)	23 (8.3)	-		
	Yes	99 (96.1)	4 (3.9)	0.44 (0.13-1.19, p=0.144)	#	0.40 (0.06-2.50, p=0.333)
Gram-negative infections	No	135 (88.8)	17 (11.2)	-		
	Yes	217 (95.6)	10 (4.4)	0.37 (0.16-0.81, p=0.015)	#	0.24 (0.03-1.99, p=0.172)
Gram-positive infections	No	258 (94.9)	14 (5.1)	-		
	Yes	94 (87.9)	13 (12.1)	2.55 (1.14-5.65, p=0.020)	#	1.00 (0.11-9.98, p=0.998)
Fungal infections	No	322 (92.5)	26 (7.5)	-		
	Yes	30 (96.8)	1 (3.2)	0.41 (0.02-2.05, p=0.393)		
CRE infections	No	329 (92.7)	26 (7.3)	-		
	Yes	23 (95.8)	1 (4.2)	0.55 (0.03-2.78, p=0.566)		
ESBL infections	No	304 (92.4)	25 (7.6)	-		
	Yes	48 (96.0)	2 (4.0)	0.51 (0.08-1.78, p=0.365)		
MDR infections	No	291 (93.3)	21 (6.7)	-		
	Yes	61 (91.0)	6 (9.0)	1.36 (0.48-3.33, p=0.522)		
MRSA infections	No	341 (92.9)	26 (7.1)	-		
	Yes	11 (91.7)	1 (8.3)	1.19 (0.06-6.50, p=0.869)		
Place of Acquisition	Community	190 (89.6)	22 (10.4)	-		
	Hospital	162 (97.0)	5 (3.0)	0.27 (0.09-0.67, p=0.009)	#	4.80 (0.57-50.13, p=0.162)
	No	254 (92.7)	20 (7.3)	-		

Cephalosporins (90-day exposure)	Yes	98 (93.3)	7 (6.7)	0.91 (0.35-2.12, p=0.830)		
Aminoglycosides (90-day exposure)	No	331 (93.5)	23 (6.5)	-		
	Yes	21 (84.0)	4 (16.0)	2.74 (0.75-7.96, p=0.086)	#	189.1 (2.47-18342.5, p=0.020)
Tetracyclines (90-day exposure)	No	336 (93.3)	24 (6.7)	-		
	Yes	16 (84.2)	3 (15.8)	2.62 (0.58-8.58, p=0.146)	#	0.35 (0.01-14.33, p=0.559)
B-lactam/B-lactamase (90-day exposure)	No	295 (91.9)	26 (8.1)	-		
	Yes	57 (98.3)	1 (1.7)	0.20 (0.01-0.97, p=0.117)	#	0.01 (0.00-0.23, p=0.013)
Macrolides (90-day exposure)	No	318 (92.2)	27 (7.8)	-		
	Yes	34 (100.0)	0 (0.0)	* (p=0.989)		
Glycopeptides (90-day exposure)	No	323 (93.4)	23 (6.6)	-		
	Yes	29 (87.9)	4 (12.1)	1.94 (0.54-5.47, p=0.251)		
Nitroimidazole (90-day exposure)	No	326 (93.1)	24 (6.9)	-		
	Yes	26 (89.7)	3 (10.3)	1.57 (0.36-4.88, p=0.486)		
Colistin (90-day exposure)	No	351 (92.9)	27 (7.1)	-		
	Yes	1 (100.0)	0 (0.0)	* (p=0.989)		
B-lactams (90-day exposure)	No	282 (91.3)	27 (8.7)	-		
	Yes	70 (100.0)	0 (0.0)	* (p=0.989)		
Glycylcycline (90-day exposure)	No	349 (92.8)	27 (7.2)	-		
	Yes	3 (100.0)	0 (0.0)	* (p=0.988)		
Oxazolidinones (90-day exposure)	No	350 (92.8)	27 (7.2)	-		
	Yes	2 (100.0)	0 (0.0)	* (p=0.990)		
90-day exposure to any antibiotic	No	201 (91.0)	20 (9.0)	-		
	Yes	151 (95.6)	7 (4.4)	0.47 (0.18-1.08, p=0.091)	#	0.28 (0.04-1.46, p=0.157)
90-days prior infection	No	268 (91.8)	24 (8.2)	-		
	Yes	84 (96.6)	3 (3.4)	0.40 (0.09-1.18, p=0.141)	#	0.21 (0.02-1.70, p=0.179)
Previous Gram-negative infection	No	307 (92.7)	24 (7.3)	-		
	Yes	45 (93.8)	3 (6.2)	0.85 (0.20-2.57, p=0.801)		
Previous Gram-positive infection	No	320 (92.8)	25 (7.2)	-		
	Yes	32 (94.1)	2 (5.9)	0.80 (0.13-2.86, p=0.768)		
Previous fungal infection	No	348 (92.8)	27 (7.2)	-		
	Yes	4 (100.0)	0 (0.0)	* (p=0.991)		
Previous SARSCoV19 infection	No	332 (92.5)	27 (7.5)	-		
	Yes	20 (100.0)	0 (0.0)	* (p=0.986)		
Polymicrobial Infections	No	140 (90.3)	15 (9.7)	-		
	Yes	212 (94.6)	12 (5.4)	0.53 (0.24-1.16, p=0.113)	#	2.68 (0.24-24.41, p=0.386)
Concurrent Gram-negative infection	No	191 (91.0)	19 (9.0)	-		
	Yes	161 (95.3)	8 (4.7)	0.50 (0.20-1.13, p=0.110)	#	0.36 (0.04-4.35, p=0.394)
Concurrent Gram-positive infection	No	252 (92.3)	21 (7.7)	-		
	Yes	100 (94.3)	6 (5.7)	0.72 (0.26-1.74, p=0.492)		
Concurrent Fungal infection	No	293 (91.6)	27 (8.4)	-		
	Yes	59 (100.0)	0 (0.0)	* (p=0.985)		
Concurrent SARSCoV19 infection	No	348 (93.5)	24 (6.5)	-		
	Yes	4 (57.1)	3 (42.9)	10.87 (2.05-52.1, p=0.003)	#	4.92 (0.04-1209.39, p=0.612)

#: variables with p-values ≤ 0.2 are enrolled in multivariate analysis, , 95% CI: Confidence intervals.

Table S4. Predictors for infection-related 28-day mortality. 13

		No	Yes	Univariable OR (CI, P)		Multivariable OR (CI, P)
Sex	F	114 (100.0)	0 (0.0)	-		-
	M	235 (88.7)	30 (11.3)	* (p=0.986)		
Age on admission Years.	Mean (SD)	57.6 (21.3)	70.8 (14.8)	1.04 (1.01-1.06, p=0.002)	#	0.96 (0.89-1.03, p=0.257)
	≤ 60 Years	185 (97.4)	5 (2.6)	-		
	> 60 years	164 (86.8)	25 (13.2)	5.64 (2.29-17.01, p=0.001)	#	66.8 (3.58-2143.2, p=0.009)
Length of stay.	Mean (SD)	26.1 (34.3)	20.7 (4.2)	0.99 (0.98-1.01, p=0.396)		
	≤ 14 days	195 (100.0)	0 (0.0)	-		
	> 14 days	154 (83.7)	30 (16.3)	* (p=0.988)		
Admission with infectious disease	No	102 (96.2)	4 (3.8)	-		
	Yes	247 (90.5)	26 (9.5)	2.68 (1.01-9.27, p=0.073)	#	4.02 (0.97-21.13, p=0.072)
Admission to critical care area	No	241 (92.0)	21 (8.0)	-		
	Yes	108 (92.3)	9 (7.7)	0.96 (0.40-2.10, p=0.914)		
Admission to medical wards	No	224 (96.1)	9 (3.9)	-		
	Yes	125 (85.6)	21 (14.4)	4.18 (1.91-9.87, p=0.001)		
Diabetes	No	118 (94.4)	7 (5.6)	-		
	Yes	231 (90.9)	23 (9.1)	1.68 (0.73-4.33, p=0.246)		
Chronic renal failure	No	144 (95.4)	7 (4.6)	-		
	Yes	205 (89.9)	23 (10.1)	2.31 (1.01-5.95, p=0.060)	#	0.02 (0.00-0.40, p=0.015)
Active malignancy	No	341 (93.4)	24 (6.6)	-		
	Yes	8 (57.1)	6 (42.9)	10.66 (3.28-33.2, p<0.001)	#	* (p=0.830)
Immunosuppressed	No	331 (93.2)	24 (6.8)	-		
	Yes	18 (75.0)	6 (25.0)	4.60 (1.55-12.15, p=0.003)	#	0.00 (0.00-1.13, p=0.843)
Chronic Cardiac Diseases	No	94 (96.9)	3 (3.1)	-		
	Yes	255 (90.4)	27 (9.6)	3.32 (1.14-14.12, p=0.053)	#	4.30 (0.39-67.64, p=0.260)
HIV follow up	No	348 (92.1)	30 (7.9)	-		
	Yes	1 (100.0)	0 (0.0)	* (p=0.989)		
Chronic Resp. Disease	No	281 (90.9)	28 (9.1)	-		
	Yes	68 (97.1)	2 (2.9)	0.30 (0.05-1.02, p=0.101)	#	0.00 (0.00-0.07, p=0.001)
Sickle Cell	No	331 (91.9)	29 (8.1)	-		
	Yes	18 (94.7)	1 (5.3)	0.63 (0.03-3.25, p=0.663)		
Other comorbidities	No	194 (97.0)	6 (3.0)	-		
	Yes	155 (86.6)	24 (13.4)	5.01 (2.12-13.79, p=0.001)	#	0.13 (0.01-1.51, p=0.117)
Comorbid conditions	Mean (SD)	3.7 (1.4)	4.8 (1.6)	1.81 (1.33-2.55, p<0.001)	#	3.16 (0.77-15.18, p=0.123)
	No	41 (100.0)	0 (0.0)	-		
	Any comorbid	308 (91.1)	30 (8.9)	* (p=0.987)		
Blood transfusion	No	162 (97.0)	5 (3.0)	-		
	Yes	187 (88.2)	25 (11.8)	4.33 (1.75-13.06, p=0.003)	#	13.70 (2.85-91.25, p=0.003)
Invasive procedure during admission	No	94 (100.0)	0 (0.0)	-		
	Yes	255 (89.5)	30 (10.5)	* (p=0.987)		
Surgery 90-day history	No	314 (92.4)	26 (7.6)	-		
	Yes	35 (89.7)	4 (10.3)	1.38 (0.39-3.80, p=0.569)		
Bacteremia	No	270 (92.8)	21 (7.2)	-		
	Yes	79 (89.8)	9 (10.2)	1.46 (0.62-3.24, p=0.362)		
Body Fluids	No	348 (92.1)	30 (7.9)	-		
	Yes	1 (100.0)	0 (0.0)	* (p=0.989)		
Respiratory infections	No	270 (93.8)	18 (6.2)	-		
	Yes	79 (86.8)	12 (13.2)	2.28 (1.03-4.89, p=0.037)	#	1.28 (0.38-4.35, p=0.689)
Skin and soft tissue infections	No	247 (89.5)	29 (10.5)	-		
	Yes	102 (99.0)	1 (1.0)	0.08 (0.00-0.40, p=0.015)	#	0.14 (0.00-1.35, p=0.148)
Gram-negative infections	No	140 (92.1)	12 (7.9)	-		
	Yes	209 (92.1)	18 (7.9)	1.00 (0.47-2.20, p=0.990)		
Gram-positive infections	No	248 (91.2)	24 (8.8)	-		
	Yes	101 (94.4)	6 (5.6)	0.61 (0.22-1.46, p=0.301)		
Fungal infections	No	323 (92.8)	25 (7.2)	-		
	Yes	26 (83.9)	5 (16.1)	2.48 (0.79-6.58, p=0.086)	#	4.35 (0.54-34.25, p=0.157)
CRE infections	No	327 (92.1)	28 (7.9)	-		
	Yes	22 (91.7)	2 (8.3)	1.06 (0.16-3.87, p=0.938)		
ESBL infections	No	300 (91.2)	29 (8.8)	-		
	Yes	49 (98.0)	1 (2.0)	0.21 (0.01-1.02, p=0.131)	#	0.38 (0.02-3.09, p=0.431)
MDR infections	No	289 (92.6)	23 (7.4)	-		
	Yes	60 (89.6)	7 (10.4)	1.47 (0.56-3.41, p=0.400)		
MRSA infections	No	337 (91.8)	30 (8.2)	-		
	Yes	12 (100.0)	0 (0.0)	* (p=0.989)		
Place of Acquisition	Community	204 (96.2)	8 (3.8)	-		
	Hospital	145 (86.8)	22 (13.2)	3.87 (1.74-9.48, p=0.002)	#	6.62 (1.44-37.62, p=0.021)
	No	257 (93.8)	17 (6.2)	-		

Cephalosporins (90-day exposure)	Yes	92 (87.6)	13 (12.4)	2.14 (0.98-4.55, p=0.050)	#	13.21 (2.3-102.04, p=0.007)
Aminoglycosides (90-day exposure)	No	324 (91.5)	30 (8.5)	-		
	Yes	25 (100.0)	0 (0.0)	* (p=0.985)		
Tetracyclines (90-day exposure)	No	330 (91.7)	30 (8.3)	-		
	Yes	19 (100.0)	0 (0.0)	* (p=0.987)		
B-lactam/B-lactamase (90-day exposure)	No	300 (93.5)	21 (6.5)	-		
	Yes	49 (84.5)	9 (15.5)	2.62 (1.09-5.91, p=0.024)	#	22.1 (4.66-137.65, p<0.001)
Glycopeptides (90-day exposure)	No	316 (91.3)	30 (8.7)	-		
	Yes	33 (100.0)	0 (0.0)	* (p=0.989)		
Nitroimidazole (90-day exposure)	No	320 (91.4)	30 (8.6)	-		
	Yes	29 (100.0)	0 (0.0)	* (p=0.989)		
Colistin (90-day exposure)	No	348 (92.1)	30 (7.9)	-		
	Yes	1 (100.0)	0 (0.0)	* (p=0.989)		
Glycylcycline (90-day exposure)	No	346 (92.0)	30 (8.0)	-		
	Yes	3 (100.0)	0 (0.0)	* (p=0.988)		
Oxazolidinones (90-day exposure)	No	347 (92.0)	30 (8.0)	-		
	Yes	2 (100.0)	0 (0.0)	* (p=0.990)		
Previous Gram-negative infection	No	305 (92.1)	26 (7.9)	-		
	Yes	44 (91.7)	4 (8.3)	1.07 (0.30-2.90, p=0.909)		
Previous fungal infection	No	345 (92.0)	30 (8.0)	-		
	Yes	4 (100.0)	0 (0.0)	* (p=0.991)		
Polymicrobial Infections	No	153 (98.7)	2 (1.3)	-		
	Yes	196 (87.5)	28 (12.5)	10.93 (3.2-68.35, p=0.001)	#	31.8 (1.97-934.33, p=0.024)
Concurrent Gram-negative infection	No	205 (97.6)	5 (2.4)	-		
	Yes	144 (85.2)	25 (14.8)	7.12 (2.88-21.48, p<0.001)	#	4.73 (0.97-34.53, p=0.081)
Concurrent Gram-positive infection	No	252 (92.3)	21 (7.7)	-		
	Yes	97 (91.5)	9 (8.5)	1.11 (0.47-2.45, p=0.796)		

#: variables with *p*-values ≤ 0.2 are enrolled in multivariate analysis, , 95% CI: Confidence intervals.
